# Aging Landscapes: Clustering Neighborhood Resources for Older Adults

**DOI:** 10.1093/geroni/igaf122.4354

**Published:** 2025-12-31

**Authors:** Eric Delmelle

**Affiliations:** Lehigh University, Bethlehem, Pennsylvania, United States

## Abstract

The neighborhoods where older adults live and interact strongly shape opportunities for physical activity, social engagement, and aging in place, yet research often examines these features in isolation rather than as holistic neighborhood types. We introduce a typology of “aging landscapes” that captures how combinations of environmental and social resources evolve over time and may support or hinder healthy aging. Drawing on a longitudinal dataset (1990–2019) at the U.S. census tract level, we compiled fine-scale measures of neighborhood resources reflecting three domains: (A) physical function (e.g., recreational facilities, greenness, walkability), (B) cognitive function (e.g., cultural and civic organizations, learning locations, hobby spaces), and (C) aging in place (e.g., health and social services, food access, third places). Variables were rescaled and reduced using Principal Component Analysis within each domain, and k-means clustering was applied to identify tract-level neighborhood types across three decades. This approach allows neighborhoods to change cluster membership over time, reflecting dynamic improvements or losses in resources. We also conduct exploratory PCA across all variables to evaluate whether data-driven structures align with theoretically defined domains. The resulting typology provides new insights into the spatial distribution and temporal evolution of resource-rich and resource-poor environments for older adults. This work provides a scalable framework for identifying and monitoring aging-supportive communities, offering new directions for urban planning and public health practice aimed at promoting environments that enable healthy aging.

